# Complex hand dexterity: a review of biomechanical methods for measuring musical performance

**DOI:** 10.3389/fpsyg.2014.00414

**Published:** 2014-05-12

**Authors:** Cheryl D. Metcalf, Thomas A. Irvine, Jennifer L. Sims, Yu L. Wang, Alvin W. Y. Su, David O. Norris

**Affiliations:** ^1^Rehabilitation and Health Technologies, Faculty of Health Sciences, University of SouthamptonSouthampton, Hampshire, UK; ^2^Music, Faculty of Humanities, University of SouthamptonSouthampton, Hampshire, UK; ^3^SCREAM Laboratory, Computer Science and Information Engineering, National Cheng Kung UniversityTainan, Taiwan

**Keywords:** musical performance, kinematics, hand, piano, expertise, virtuoso, novice, health

## Abstract

Complex hand dexterity is fundamental to our interactions with the physical, social, and cultural environment. Dexterity can be an expression of creativity and precision in a range of activities, including musical performance. Little is understood about complex hand dexterity or how virtuoso expertise is acquired, due to the versatility of movement combinations available to complete any given task. This has historically limited progress of the field because of difficulties in measuring movements of the hand. Recent developments in methods of motion capture and analysis mean it is now possible to explore the intricate movements of the hand and fingers. These methods allow us insights into the neurophysiological mechanisms underpinning complex hand dexterity and motor learning. They also allow investigation into the key factors that contribute to injury, recovery and functional compensation. The application of such analytical techniques within musical performance provides a multidisciplinary framework for purposeful investigation into the process of learning and skill acquisition in instrumental performance. These highly skilled manual and cognitive tasks present the ultimate achievement in complex hand dexterity. This paper will review methods of assessing instrumental performance in music, focusing specifically on biomechanical measurement and the associated technical challenges faced when measuring highly dexterous activities.

## INTRODUCTION

A functional hand is capable of performing a wide variety of activities, from writing and eating, through to communicative gesture and interactions with the environment around us. This expansive repertoire facilitates a vast, almost limitless collection of task objectives and requires a versatile portfolio of movements in the wrist and hand allowing us to achieve success. The versatility inherent in this repertoire is possible due to the integration of the physical musculoskeletal structure (including an opposing thumb) and a highly evolved neurological and sensorimotor control system.

The acquisition of skill has been studied from many perspectives, including from an evolutionary perspective (Marzke and Marzke, 2000; Olivier et al., 2007), through the process of learning (Watson, 2006; Olivier et al., 2007), recovery from injury or deterioration, and compensation resulting from illness (Butler and Rosenkranz, 2006). There are many methods available to advance knowledge in this area, such as imaging techniques (Lotze et al., 2003; Gibson et al., 2009), and these have been utilized to describe the neurophysiology and motor control principles of the hand. Campbell (1905) first described the human motor cortex map and its role as the primary region of the cerebral cortex involved in planning, control and execution of voluntary movements, especially in musical performance (Bangert and Altenmüller, 2003; Kristeva et al., 2003). Understanding these cognitive processes that contribute to performance is important to further our understanding of the mechanisms that underpin complex hand dexterity. Therefore the following section will provide a brief overview of these fundamental, neurophysiological systems.

### COGNITIVE ASPECTS OF COMPLEX HAND DEXTERITY

Performance of any skilful activity is regulated by a highly refined and integrated system that includes motor planning, sensorimotor integration, execution and adaptation, following either disruption or improvisation. A complex, integrated feedback loop is created by receiving information from sights and sounds, and by interacting with the external environment (the music, the orchestra/other musicians, the conductor, and the instrument). The ability to harmonize a physiological response to changes in environmental alterations relies on the fluid integration of multisensory stimulus and appropriate physical adjustments in motor control.

Motor control is a learned skill that is continually developed throughout one’s life. The success of a planned motor response will be determined by a number of factors. These factors include motivation and goal-identification, experience of the same or similar tasks, success and rewards. Underpinning these cognitive processes is a complex omnidirectional network. This comprises multisensory inputs via afferent feedback from the environment, for example visual, auditory and somatosensory (both tactile and kinaesthetic, see Demain et al. (2013) for further information). Sensory information is then integrated depending on the intention of the task, for example integration of the visual and proprioceptive (joint positions) senses to estimate a movement trajectory toward a target (piano keys). Sensory information then enters the cerebral cortex, which includes the motor cortices; premotor, motor and supplementary cortices (Enoka, 2008; McMillan and Carin-Levy, 2012).

Motor activity originates in the motor cortices, basal ganglia and cerebellum. Voluntary and automatic movements are initiated in the motor cortex and basal ganglia respectively. The cerebellum integrates vestibular, visual, proprioceptive and tactile sensory information, and by using this integrated information, adjustments can be made to cortical output to modify the amplitude and trajectory of movement, for example when you react to a perturbation in the environment (Enoka, 2008). Adjustments to the movement are auctioned via descending neural pathways, including the corticospinal tract. In addition, the vestibulospinal and reticulospinal tracts, arising in the brain stem, ensure the appropriate postural tone in the trunk and shoulder girdle, thus stabilizing the upper limb and allowing flexible control of the wrist and fine, dexterous movement of the fingers.

Signals then travel via the upper motor neurones, which terminate on the anterior horn cells in the spinal cord. Action potentials generated in the anterior horn cells are conducted via lower motor neurons, which synapse on the muscles and produce a muscle contraction (McMillan and Carin-Levy, 2012). The strength of the muscle contraction depends on both the number (spatial summation) and rate (temporal summation) of lower motor neurone activation. Finally, further control of anterior horn cells output is achieved through direct sensory input at spinal cord level, such as what occurs in a tendon reflex.

The cognitive, sensorimotor and neurophysiological elements involved in the preparation, control and execution of a functional movement have been well defined in the literature, and therefore to review these in depth would be supplementary to the primary focus of this review. Therefore, for more information on these areas, refer to Jeannerod (1997), Peretz and Zatorre (2003), Jones and Lederman (2006), and McMillan and Carin-Levy (2012).

### IMAGING

With specific reference to measuring musical performance, imaging studies have been used to identify cortical activity. During magnetic resonance imaging (MRI) and magnetoencephalographic (MEG) studies, professional musicians (compared to amateur and non-musicians) showed a greater volume of gray matter and/or enlarged cortical representation in the primary motor, somatosensory and premotor areas, the cerebellum, and the anterior superior parietal areas, thus illustrating honed ability in sensorimotor control, audio-visual and spatial processing (Elbert et al., 1995; Schlaug, 2001; Münte et al., 2002; Gaser and Schlaug, 2003; Hutchinson et al., 2003). In addition, Bengtsson et al. (2005) used diffusion tensor imaging to study the how piano practice affects white matter between three different age groups (children, adolescents and adults). The results showed that within each age group, practice was positively correlated with fiber tract organization in different regions. These results are interesting because they indicate structural differences in the musical brain: a powerful motivation for increasing the potential for intellectual endeavor. With reference to the study by Bengtsson et al. (2005), the results also suggest that the training undertaken by a practicing pianist while the fiber tracts are maturing induce white matter plasticity. Thus an increase in activity, initiated by piano practice, causes increased myelination (the increase of myelin around axons of neurons). For a detailed review on neuroimaging of structural plasticity see Zatorre et al. (2012).

It is at the level of cortical organization that the relationship between complex hand dexterity and bimanual instrumental practice can be observed. Elbert et al. (1995) showed increased cortical representation of the left hand (fingering hand) in string players versus the right hand (bowing hand); indicating the musician’s choice of instrument influences changes at a cortical level, and thus makes findings less generalizable to “musicians” *per se*. This review will specifically concentrate on piano performance, where bilateral fine motor control is compulsory. For more information on imaging techniques and reviews on neuroplastic changes underpinning motor control and learning, see Münte et al. (2002), Watson (2006), and Furuya and Altenmüller (2013).

Despite this interest, our understanding of complex hand dexterity is still in its infancy. Fundamental questions still remain; particularly surrounding how function is honed in various activities for virtuoso expertise to manifest. The multidisciplinary nature of this area brings together neuroscience, motor learning and control, physiology, biomechanics, anthropology, behavioral, and cognitive sciences. This narrative review will focus on the relatively new use of biomechanical methods and their emerging contribution to this subject, given the complex nature of analysis required. This review presents a comprehensive background section giving an overview of complex hand dexterity (See The Human Hand in Performance). This is followed by an historical overview of the pioneering techniques developed for capturing movement (See Historical Methods for Capturing and Measuring Movements) and current biomechanical methods, derived from those techniques, to measure complex hand dexterity (See Current Biomechanical Methods of Measuring Complex Hand Dexterity During Musical Performance).

## PART 1: THE HUMAN HAND IN PERFORMANCE

### COMPLEXITY IS COMMONPLACE: INTRODUCING COMPLEX HAND DEXTERITY

The versatility of possible hand movements available to complete any given task has made standardization of activity difficult, therefore hindering comparative investigation (Metcalf, 2009). In addition, the complexity of movements and the smaller anatomy of the hand have historically limited our ability to measure all the composite movements of the wrist, hand, fingers and thumb. Hand movements provide a conduit for examining how skill manifests at a physical and actioned level. It is the result of intention.

**FIGURE 1:**
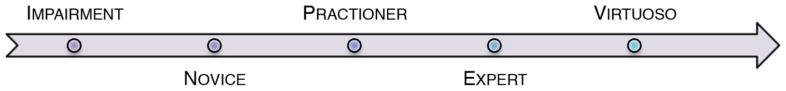
**Skill acquisition as a spectrum of complex hand dexterity**.

Hand dexterity can be thought of as a spectrum (see **Figure 1**); from impaired, affected function through to highly skilled, virtuoso performance. Complex hand dexterity describes the skill(s) that are required for increasingly higher levels of function. Thus a virtuoso performer will exhibit the highest levels of complex hand dexterity in their given activity.

Musical expertise is extraordinary. The speed, fluency and tempo of elite instrumentalists can be mesmerizing to witness and often audiences will favor seats where they can observe feats of seemingly impossible dexterity and control during a performance. Musical expertise is arguably the ultimate example of elite performance in complex hand dexterity. Aspects of motor control and learning, precision, timing, strategies for compensation, adaptation and coordination can be observed during playing. Analysis of instrumentalists allows us to study complex hand dexterity just as the analysis of elite sports allows us to study whole-body dynamics.

### MOVEMENT VARIABILITY AND REDUNDANCY: “LIVE MOVEMENT IS A BALL OF ENTANGLED INTERACTIONS” (Bernstein and Popova, 1930)

Glazier et al. (2006, p. 50), stated “movement variability is an essential feature of human motor behavior that affords the sensorimotor system the necessary flexibility and adaptability to operate proficiently in a variety of performance, development and learning contexts” (Bernstein and Popova, 1930). The literature widely supports this statement and, particularly with reference to the upper limb, is an essential factor in allowing the versatility of movements required to perform functional activities. This versatility has also been referred to as the “degrees of freedom problem” by Bernstein (1967b). He commented on the redundant degrees of freedom (DOF) observed in the upper limb, and how this facilitates the complex range of solutions to everyday activities. Bernstein also commented on the human capacity to hone the multiple and redundant DOF in the upper limb to facilitate highly skilled activities, such as musical expertise (Bernstein and Popova, 1930).

Manifestation of skill can therefore be observed and quantified by the analysis of movement. Studying movement allows analysis of the functional strategies adopted by an individual to achieve a goal, whether that goal is playing a piano to produce “musically beautiful sounds” (Bernstein, 1967a), holding a teacup or using a tool.

The following section will review the historical techniques for capturing and measuring movements during the late nineteenth/early twentieth century and provide the precursors for current biomechanical methods of measuring complex hand dexterity during musical performance.

## PART 2: HISTORICAL METHODS FOR CAPTURING AND MEASURING MOVEMENTS

### LATE 19TH AND EARLY 20TH CENTURY EXAMPLES OF CAPTURING MOTION

Modern methods of optical motion capture can trace its origins to the late nineteenth century and the pioneering work of individuals such as Marey (1873), Muybridge (1887), and Braune and Fischer (1895). The enthusiastic adoption of the newly developed field of photography, and particularly sequence photography, allowed human and animal movement to be not only captured, but analyzed for the first time.

In 1878, a photographer, Eadweard Muybridge, devised an experiment whereby a horse would gallop through a track lined with still photographic cameras. These individual cameras were triggered in succession as a horse galloped down the track by threads placed along the width of the track and attached to the camera shutters; capturing the movements of the gallop. Muybridge then “played” the silhouette of each image in sequence using a Zoopraxiscope. This method was groundbreaking and considered to be the first movie projector.

By 1884, Etienne-Jules Marey, a physiologist, had designed a black suit with white tape down the arms and legs, which was to be the first method of marker-based motion capture. Marey also revolutionized the ability to capture motion; developing systems capable of capturing 12 frames per second, and then later 60 frames per second (Klette and Tee, 2008).

With the formation of the motion picture industry during the early 20th century, motion analysis became a useful tool that was adopted in various ways from scientific to artistic pursuits. Concurrently, in 1927 Nikolai Bernstein, a neurophysiologist, brought a new scientific approach to studying human movement and based his hypotheses in neurophysiology and motor control. Münte et al. (2002) and Furuya and Altenmüller (2013) have also adopted this approach much later. Bernstein et al. also developed a new way of capturing motion, the Kymocyclograph, which has been heralded as “probably unsurpassed until the recent advent of optoelectronic techniques” (Kay et al., 2003). The Kymocyclograph used film and a camera shutter lens at high speed to capture images of light bulbs placed on the moving body (Kay et al., 2003). The Kymocyclograph was capable of capturing up to 600 images per second and presented a series of images of the light bulbs at positions throughout a movement. Bernstein et al. used these images to measure joint movement (Gelf and Latash, 2002). The combination of this new technology, and rigorous methods of analysis, forged a new area: the field of biomechanics.

Bernstein was a proponent of biomechanics and advocated it as a tool for understanding complex interactions of sensorimotor system using robust, accurate and objective measures. One application area investigated by Bernstein and his team was the “biodynamics of piano strike” (Bernstein and Popova, 1930). In one of the earliest examples of studying musical technique using biomechanical methods, Bernstein used his Kymocyclograph to answer the questions: (1) “Do changes in the tempo of a movement influence its construction and dynamics?” and (2) “To what extent does the weight of the extremity contribute to the studied exercise?” The study compared shoulder, elbow and wrist movement of pianists and selected music that constrained the movements of the individual to eliminate any opportunity for “artistic performance.” The results of this early study showed inter-joint coordination increases with increased tempo, where faster tempi show the movements that contribute to key strike become more continuous and fluid, and slow tempi appear segmented. This had interesting implications in piano pedagogy by identifying that coordinated movement sequences are fundamentally different when played at different tempo. This indicates that from a motor control perspective, practicing a piece of music at a slower pace does not help learning because as speed increases, old movement sequences are un-learned and new sequences learned. It is therefore beneficial to practice at the required tempo from an early stage of learning each piece. However, this is not the only perspective and from a pianist’s perspective, note learning and memorization may require slow practice.

This study also showed that at faster tempi, wrist movement seemed “forced” by elbow movement, thus illustrating for the first time that these adjoining segments are highly coupled. This has interesting implications for motor learning and automatic sequence actuation. Bernstein and Popova (1930) also clarified that the weight of the extremity does not contribute to an exercise, as active muscle forces are present during movement illustrating independence.

The following section will provide an overview of the advances in motion analysis and where appropriate, will provide examples of their application within musical performance.

### HISTORICAL DEVELOPMENT OF MOVEMENT ANALYSIS

Within biomechanics, arguably the most common and widely accepted methods of motion capture are camera-based optoelectronic systems. As this technology has advanced, so has the ability to study movements in greater detail. Throughout the latter part of the 20th century the majority of motion capture was applied to analysis of gait. Walking could be simplified into a two-dimensional (2D) perspective. Providing information from the sagittal plane allowed study of the primary components of gait, i.e., hip and knee flexion/extension and foot dorsiflexion/plantar flexion. The assumption that gait can be simplified into a 2D perspective obviously affects the accuracy of the measurement. In simplifying to two-dimensions, any third-dimensional rotations are lost; rotations are often affected during injury and impairment and omitting these characteristics limits useful measurement.

However, due to the complex anatomy and vast movement potential within the upper limb (Bernstein’s redundancy), methods of motion capture are required to be comprehensive, three-dimensional (3D) and versatile. Until recently, camera-based motion analysis of the distal upper limb, namely the wrist and hands, were limited (Metcalf, 2008); thus giving rise to alternative methods of motion capture, with inherent benefits and limitations.

Glove-based systems allow analysis of movements that may otherwise be occluded using camera-based technology. However the accuracy of glove-based systems has been shown to vary greatly. Overall average errors of 11°; have been reported when compared to computed tomography (CT; Buffi et al., 2011), which are higher than figures shown for goniometry of the hand; the gold standard for clinical-based joint range of motion measurement (7–9°; Ellis et al., 1997; Ellis and Bruton, 2002). Errors at specific joints varied from 1 to 23°, generally increasing in more distal joints, specifically the distal interphalangeal (DIP) joint (9–23° error), which limits interpretation of any results (Buffi et al., 2011). The technical rationale behind such errors is probably due to the technology that underpins these systems. The resistive flex sensors are placed in the glove on the dorsal aspect of the hand. Movement of the glove material, and therefore the sensors, is independent of the underlying joints, therefore increasing the potential for measurement error. In optical marker-based motion capture, there is always an error due to skin movement. In glove-based systems, this error is compounded due to the additional movement of the glove material. In addition to being less accurate than optical motion capture systems, glove-based systems can, by their very nature, impede movement of the joints, just as when wearing a normal glove.

The type of glove used will also have an impact on the accuracy of measurement. Such as the CyberGlove II, this uses two sensors per finger, therefore omitting movement of each DIP joint. In addition, many glove systems cover the fingertips, thus affecting tactile feedback from the piano keys. An important aspect of motor learning is sensory input to an integrated sensorimotor system. Tactile feedback from keys plays an important role in feedback for learning and memory recall. Goebl and Palmer (2008) showed that tactile feedback from key press enhances timing accuracy through practice. Sensorimotor feedback should not therefore be underestimated. For a comprehensive overview of the anatomy and physiology of the tactile sensory system, see Demain et al. (2013).

Glove-based systems, however, have been used for measurement of hand kinematics during instrumental performance (Furuya et al., 2011a). In this study, the authors used Principal Component Analysis to identify patterns of coordination and synergistic responses between individual movements of the hand. The effect of tempo on finger movements was also investigated in a later analysis of this study (Furuya and Soechting, 2012), and will be discussed later in Section “Movements at Faster Tempi.”

Other methods of motion capture were also trialed in the analysis of pianist movements, namely inertial measurement devices, such as accelerometers. Sensor topology conforming to the underlying anatomy resulted in individual units being applied to each phalanx of the finger (Rahman et al., 2011; Kortier et al., 2012). The large size of these devices provides a distracting and impeding obstruction to “natural” performance, which may contribute to these sensors not being widely adopted. In addition, these devices are tethered to provide power and data transfer, which can also hinder performance. Tethering can also be observed in some optical motion capture systems, such as the CODA system (Rahman et al., 2011).

As technology progressed, so did the incentive to investigate hand and wrist movements. An important consideration when using any measurement system is that you do not change the natural process of what you are trying to measure. This is particularly relevant when assessing the complicated, intricate movements involved in complex hand dexterity. While optical motion capture was still predominantly applied in gait analysis, pointing, reaching and grasping analysis did not begin in earnest until the 1980s (Jones and Lederman, 2006). However, advances in optical camera technology, from approximately 2000, were integral to methods of hand movement analysis being developed and adopted; enabling research on complex hand dexterity. Analysis of complex hand dexterity will further our understanding in areas of motor learning, skill acquisition, intention and virtuoso performance; facilitating an approach to understanding motor control.

These analyses however require a much higher level of precision. This poses the problem of balancing the need for optimal data collection through system set up and any imposed physical restrictions from associated equipment, whilst allowing for functional, natural movements. In order to investigate any kind of movement performance using biomechanical techniques, natural movements must be allowed in order to analyse true performance.

In contrast to other systems, marker-based motion capture systems are highly accurate but also have potential limitations, such as marker placement error and accuracy due to marker occlusion (Metcalf, 2009). This occlusion can be overcome, to some extent, by the use of algorithms that utilize various methods of interpolation.

An alternative to marker-based systems is markerless systems: systems that rely on tracking algorithms and landmark definition to identify the hand and its segmental anatomy. Topical interest in adapting gaming technology to capture and measure body movement is increasing. Researchers are now concentrating on capture and measurement of hand and finger movements (Metcalf et al., 2013). These systems are versatile in their potential application and do not require any additional setup equipment, in contrast to their marker-based and glove-based counterparts, however they are limited by their reduced accuracy.

The following section will review how various current methods have been used to quantify complex hand dexterity during musical performance. Each section will highlight studies and, where necessary, describe results that provide important information for this emerging field.

## PART 3: CURRENT BIOMECHANICAL METHODS OF MEASURING COMPLEX HAND DEXTERITY DURING MUSICAL PERFORMANCE

### BIOMECHANICS IN PERFORMANCE SCIENCE

The following section will outline the adoption of various biomechanical techniques to further our understanding of complex hand dexterity and how they have been adopted within the study of musical performance. A comprehensive approach is required to provide a neurophysiological roadmap of complex hand dexterity. However many studies only focus on particular aspects of this problem, such as particular joint movements, muscle activity, motor cortex activation through movement initiation, etc., probably due to the inherent complexity of the activity and the volume of data involved. To date, there is no comprehensive study that takes into account all these neurophysiological parameters.

#### Movements at faster tempi

In piano playing there is a direct relationship between key press velocity and tone intensity. Subtle changes in the musical score require the performer to respond at a kinematic level, through adjustments in tempi and force, to produce the tones required of the piece.

Some researchers have used electroencephalography (EEG) to measure the electrical activity across the surface of the brain. This technique can be used to assess which areas of the brain (i.e., cortices) are active during performance. EEG can provide a cheaper alternative to the aforementioned imaging techniques and can be a powerful tool for understanding the origins of functional movements and mechanisms of feedback, which is intrinsic to performance. Researchers have used EEG to reveal that event-related potentials (i.e., brain activity) are affected by a change in tempo (Jongsma et al., 2007; Yuan et al., 2009).

Goebl and Palmer (2009) investigated changes in dynamic finger kinematics with changes in tempo. In their study, height of the finger above the piano key (given by marker motion trajectories) was analyzed with changing tempo. The results showed more controlled motion trajectories at slower tempi and increasingly erratic trajectories to accommodate faster tempi. In a similar protocol where markers were placed on the fingertip, Dalla Bella and Palmer (2011) showed that faster tempi resulted in greater maximum finger height above the piano keys prior to key press. These results seem to indicate the force of the movement is produced by taking advantage of the lever arm from the metacarpophalangeal (MCP) joint of the finger and this lack of motion control contributes to the erratic trajectories previously observed by Goebl and Palmer (2009). However, without detailed information on the kinematic chain, no inference can be made about the origin of the movement in the study by Della Bella and Palmer; movement originating at the MCP, contributing the finger height, or movement originating at the proximal joints (elbow). The use of proximal joints has also been shown as an indicator for expertise. Furuya et al. (2011b) studied expert and novice pianists using a Mac3D motion capture system and a two-channel electromyography (EMG) system. The results showed expert pianists reduce muscle activity and utilize more proximal joints and hand posture configuration resulting in reduced biomechanical effort.

In a later study using the CyberGlove, Furuya and Soechting (2012) studied joint velocity co-variation between fingers involved and not involved in key press. They found no difference in independent finger movements at the MCP and proximal interphalangeal (PIP) joints with increased tempo. These results were also confirmed in a later, comprehensive kinematic study, by Goebl and Palmer (2013) who investigated hand movement efficiency and joint velocity in musical performance using a Vicon motion capture system and a MIDI keyboard. Keystroke efficiency, defined by the relationship between precision in timing of tone onsets and force measurement, and individual joint contributions, were also analyzed in this study during piano playing at fast tempi. The results found that keystroke efficiency is required for achieving fast performances. This was illustrated by stability in keystroke timing, force measurement and individual joint contributions. Furuya et al. (2013) also assessed speed using a MIDI keyboard to study whether practicing movements at a submaximal speed influenced the maximum speed of finger movements during piano performance. Progressively faster speeds were observed and these maximum speeds were maintained for 2 months after assessment.

In everyday skill acquisition, there is often a tradeoff between speed and accuracy. However in practiced, memorized musical performance, there is evidence that errors do not increase with faster performance (Palmer and van de Sande, 1993, 1995; Drake and Palmer, 2000). This suggests that the high level of practice, synonymous with musical performance, produces higher levels of accuracy than other fields of manual skill development. This was however replaced with a tradeoff between relative timing and pitch, with a bias toward timing over pitch.

#### Kinematic strategies through inter-joint coordination

Fluid inter-joint coordination produces kinematic strategies that facilitate skilled performance. The ability to repeat a movement, observed at a kinematic level, has been linked with functional ability in everyday activities (Metcalf, 2008). Therefore functional ability is inversely proportional to variability in motor control. The adoption of repeated kinematic strategies therefore provides an opportunity to observe the process of learning from a bottom-up perspective. Within musical performance, independent finger movements at fast tempi are indicative of skill (Furuya and Soechting, 2012). Engel et al. (1997) also found that there is an anticipatory change of sequential hand movements in pianists that contribute to skilful execution of complex movement strategies.

Previous work examining kinematic strategies focused on posture and gross movement of the kinematic chain, often focusing on angular velocity rather than strategies of movement (Furuya et al., 2008). For example, MCP, wrist, elbow, shoulder and hip movement were analyzed by Furuya et al. (2008) using a 2D LED motion capture system. This study showed that music played at faster tempi resulted in a decrease in angular velocity of the distal joint (wrist), while the angular velocity of the proximal joints (shoulder and elbow) remained unchanged. This study also showed that there was an exponential increase in the range of movement across all joints with louder playing. These results indicate that movements of proximal joints are used to produce louder sounds, but they are used to a lesser amount when playing at faster tempi. These results are contradicted by the authors’ later work on increasing tempi of tremolo as they state the movement originates at a proximal joint (elbow) for forearm pro-/supination with less movement at the fingers (Furuya et al., 2011b). However, the very strict biomechanical criteria required to produce the tremolo movement is most likely the cause of this contradiction, rather than being a direct contradiction of the generalizable kinematic strategy.

The analysis of musical expertise allows a framework for assessing kinematic strategies and the development of “technique” honed through intensive practice. To examine this, many studies utilize motion analysis, sometimes coupled with analysis of EMG. A comparison in muscle activity between expert and novice pianists has been studied (Lai et al., 2008; Furuya and Kinoshita, 2008a). Lai et al. (2008) suggests that professional musicians effectively activate the proximal muscles to optimize movements during performance. This coincides with a reduction in coactivation at the distal joints (Furuya and Kinoshita, 2008a). The physiological response is therefore to minimize the effort required of the distal joints, which also limits fatigue in these joints. There is also preliminary evidence to suggest that the neurophysiology of expertise manifests at the level of motor unit activation. Lai et al. (2008) recorded motor unit action potentials (MUAPs) of the first dorsal interosseous muscle at successive points of a maximum voluntary contraction between pianists and non-musicians. The results illustrated that pianists produced faster responses by recruiting motor units at higher frequencies and over a shorter duration. This could potentially influence fine finger control. Several EEG studies have investigated the ability to individually control finger movements during piano performance by measuring the cortical activity in the motor regions of the brain (Slobounov et al., 2002; Chiang et al., 2004).The ability to individually control fingers is shown to be notable in expert pianists and was evident as larger electro-cortical activation when compared to non-musicians (Slobounov et al., 2002).

In an extension of previous work, Furuya and Kinoshita (2008b) used a 2D LED motion capture system to analyse inter-joint coordination and “angle of attack” (angle of the vector defined from MCP to fingertip relative to the key) during key press. Participants were required to execute simultaneous staccato touch of the G3 (thumb) and G4 (little finger) using the right-hand. These keystrokes were played at four sound levels: *piano, mezzo-piano, mezzo-forte* and *forte*. At a kinematic level, experts were shown to exhibit shoulder flexion immediately prior to key press, which highlights movement being initiated at the proximal joints. In addition, during key press, the shoulder, wrist and MCP joints (assumed as a rigid body with no PIP or DIP joints) showed simultaneous flexion with notable flexion of the wrist and MCP joint, while the elbow extended. In contrast, novice players exhibited strategies of shoulder extension that continued beyond the point of finger contact with the keys and predominantly used wrist and elbow extension and no notable wrist and MCP flexion during key press. The angle of attack was also shown to be larger in experts than novice players. The results of experts in this study can be directly compared to Goebl and Palmer (2013), whereby flexion of the MCP joint continued to the end of the key press. However, while Furuya and Kinoshita (2008b) assumed a rigid body from the MCP to the fingertip, thus omitting the PIP and DIP joints, the study by Goebl and Palmer (2013) show simultaneous extension of the DIP joint toward the end of key press motion. The PIP joint remained in flexion with little dynamic change throughout the key press. The wrist however did move from extension through to flexion. Goebl and Palmer (2013) investigated hand movement efficiency in piano playing, and particularly interdependencies of finger movements during key press (see Movements at Faster Tempi for discussion relative to tempo). A MIDI keyboard and a Vicon motion capture system were used to measure wrist and finger movements. They also captured a cyclical melody for the right hand, which may account for the difference between their results and those of Furuya and Kinoshita (2008b). In terms of the system, this technology has been proven to be the most robust and accurate (Metcalf, 2008, 2009; Metcalf et al., 2008, 2013; Metcalf and Notley, 2011), however there is no detail given on the validity of the chosen kinematic measurement technique.

This study also highlighted the importance of tempo, particularly relevant was the ability of the performer to play at faster tempi and the relationship this had to inter-joint coordination (Goebl and Palmer, 2013). In contrast to previous studies that did not study finger movements in depth (Furuya and Kinoshita, 2008b), Goebl and Palmer (2013) showed that faster movements originated at the MCP joint, with little movement at the PIP and DIP joints, whereas during slower movements, the MCP joint flexed notably followed by extension of the PIP and DIP joints.

Furuya et al. (2011b) also highlights the use of proximal joints (elbow) to reduce the extrinsic muscle activity of the fingers, thus providing a kinematic strategy that reduces overall biomechanical effort. This strategy is commonplace in other skilled activities, such as throwing (Putnam, 1993; Gray et al., 2006), where the movement originates at the shoulder and the ballistic nature of the activity provides power to the end-point of the kinematic chain, i.e., the hand. Therefore preliminary evidence suggests that some generalized principles of motor learning in other activities can be applied to more complex activities, such as musical performance. Further work is needed to establish this assertion however.

Furuya et al. (2011a) used a CyberGlove synchronized with a MIDI keyboard to describe the kinematic strategies of striking and non-striking fingers; showing similar movement covariation. The authors asserted this as evidence of independent control of finger movements (Furuya and Altenmüller, 2013) commenting on the previous work of Engel et al. (1997). This idea was further developed using a 7-channel EMG analysis of finger flexors/extensors and thumb flexor and abductor muscles. The results show evidence of coarticulation, or anticipatory control, throughout the sequential movement of the fingers (Winges et al., 2013). However, this is in direct contrast to other biomechanical studies in functional reach-to-grasp activities, where during grasp phase, movement is led by the fourth (ring) and fifth (little or pinkie) fingers, which are used as stabilizers of the object upon grasping (Zatsiorsky et al., 2000) and followed by the third (middle) and second (index) fingers, which provide the requisite power to the grasp. Parlitz et al. (1998) also showed that novices were unable to independently control movement and force of non-stroking fingers. The assertion of independent finger control in pianists (expert and novice) is inconclusive and therefore requires further investigation.

Furuya et al. (2012) analyzed finger and thumb movements of the right hand during piano playing using Principal Component Analysis to describe the patterns of movement coordination in the hand. The results showed that movements involving the thumb during playing had associated coupled movements across the other fingers at the MCP and PIP joints. In further analysis of this study, Furuya and Soechting (2012) described the variation in speed during independent control of finger movements. These results were previously described in Section “Movements at faster tempi.”. For a comprehensive overview of these works, see Furuya and Altenmüller (2013).

As current methods of data capture have improved (primarily through the adaptation of commercial gaming products), new methods are developed. MacRitchie and Bailey (2013) developed a method of using a monocular camera-based system to measure finger and thumb joint movement. The system uses ultraviolet (UV) painted markers directly on the skin of the pianist’s hands; different colored paint was used to distinguish each hand. A background UV light was used to highlight the paint on the video footage. A kinematic measurement technique was then developed to measure movement of the fingers and thumbs, including 3D estimation. The system was assessed for tracking ability (ranging from 63 to 88% in the final system) and 3D estimation (maximum variability = 2.64%). While this system lacks the comparative accuracy of laboratory-based systems and the authors comment on its practical and performance limitations, it is an exciting development with potential for applications outside strict laboratory-based applications. This opens up the potential for field observations and provides a cheaper alternative method if the limitations are deemed acceptable.

### ANALYSIS OF EXPERTISE

Research shows that healthy individuals, given an everyday task, will repeat movements with little kinematic variability (Palmer, 1997; Parlitz et al., 1998; Shan and Visentin, 2003; Metcalf, 2008). In addition, individuals with neurological dysfunction, but with functional ability, aim to minimize variability between repeated hand and wrist movements, compared with lower functioning individuals. However, as a group, impaired individuals adopt different strategies from each other (Metcalf, 2008). These observations were also shown in a healthy group (*Ibid.*). The ability of individuals to hone particular movements is therefore indicative of higher functioning, and thus indicative of developing expertise; results that have also been shown in coordination and performance in elite sports (Wagner et al., 2012). For the first time, it is possible to test performance strategies, such as minimizing variability, of higher functioning, musical virtuosos, using the most accurate and comprehensive performance metrics. Learning a strategy helps us cope with the redundant DOF in the upper limb (Davids et al., 2007). Bernstein (1967a) stated improved motor skills are synonymous with increasing the number of joints employed; utilizing more joints and less overall muscle activity, an individual becomes increasingly economical at a physiological level. Highly skilled individuals, such as musicians, can exploit the redundancy in DOF of the movements in the upper limb; reversing Bernstein’s “problem.” Redundant DOF not only provide multiple solution strategies to completing a functional task, but can also be utilized by experts to develop and hone complex hand dexterity and refine a musical piece. It can also be used for compensation during learning and adaptation following injury, pain, or impairment.

Hand function can be thought of as a spectrum of acquired skill, initially ranging from novice to expert. Individuals can be placed at either end of the spectrum; either impaired function prior to novice, or virtuoso exceeding the skill of experts. Previous research has shown that expert pianists reduce muscle activity by employing more joints proximally (Furuya and Kinoshita, 2007). It is also widely shown that brain structures and function differ between trained musicians and non-musicians as demonstrated in imaging and EEG activity studies (Rüsseler et al., 2001; Gaser and Schlaug, 2003; Limb, 2006). These factors, as well as those identified above, contribute to skill acquisition in expert musical performance.

Analysis of expertise has been the focus of many studies on musical performance. These studies identified characteristics that highlight key factors associated with, and contributing to, complex hand dexterity. The bulleted list below summarizes the findings of biomechanical studies investigating complex hand dexterity in musical performance. This list specifically summarizes the factors attributable to expert playing, and has been produced for ease of identification and further investigation.

#### Experts exhibit

##### *Force*.

• Less force required to maintain key depression for the same period of time (Parlitz et al., 1998).

##### *Speed*.

• Less time spent with fingers in contact with the key in order to produce the same note as a novice player (Parlitz et al., 1998);
• An increase in tempo that is inversely proportional to the range of joint movement (Furuya et al., 2008);
• Faster forearm pronation/supination and slower finger movement during tremolo (Furuya et al., 2011b);
• Submaximal speed facilitates fast finger movement (Furuya et al., 2013);
• Stability in timing facilitates accurate, fast performance (Drake and Palmer, 2000; Goebl and Palmer, 2013);
• A tradeoff between relative timing and pitch (Drake and Palmer, 2000).

##### *Muscle activity*.

• MUAPs recruited for the intrinsic muscles of the hand have a higher firing rate and high frequency for a shorter duration (Lai et al., 2008);
• Less co-activation of extrinsic finger muscles (Furuya et al., 2011b);
• Reduced finger muscle loads (Furuya et al., 2011b).

##### *Kinematic strategies*.

• Proximal to distal movement strategies (Putnam, 1993; Furuya and Kinoshita, 2007);
• Independent finger movements at fast tempi (Aoki et al., 2005; Furuya and Soechting, 2012);
• Stability in individual joint contributions facilitates fast performance (MacKenzie et al., 1983; Goebl and Palmer, 2013);
• Observable inter-joint coordination in the distal joints (Furuya et al., 2011b);
• Utilize proximal joints to reduce biomechanical effort (Furuya et al., 2011b);
• Shoulder flexion initiated momentarily before the finger makes contact with the key (Furuya and Kinoshita, 2008b);
• Shoulder, wrist, and finger joint flexion simultaneously, while the elbow extends during key depression (Furuya and Kinoshita, 2008b);
• Large angle of attack (Furuya and Kinoshita, 2008b).

##### *Cognitive neuroscience*.

• An ability to anticipate future plans of action (Palmer and Drake, 1997);
• Anticipatory change (coarticulation) in sequential movements of the distal joints (Engel et al., 1997).

### CONSIDERATIONS FOR HEALTH IN PERFORMANCE SCIENCE

Integrated and highly refined sensorimotor feedback is pivotal to achieving excellence in musical performance. Adopting a bottom-up approach using biomechanical methods, the functional mechanisms that contribute to the effectiveness of the sensorimotor system can be measured. Bernstein and Popova (1930), and later reignited by Münte et al. (2002) and Furuya and Altenmüller (2013), proposed that musical performance provides a unique model for assessment of motor control and learning. Furthermore, dysfunctional mechanisms, such as those following injury or disease, can also be assessed using this paradigm, thus highlighting the importance of skill acquisition as a spectrum of complex hand dexterity. There may also be additional principles that can be applied to the general population, given the high prevalence of work-related musculoskeletal disorders, such as repetitive strain, that are increasing with the use of computer keyboards (Kryger et al., 2003), and other physical interfaces, such as gaming consoles, cellular, and smartphones.

Within elite performers, such as instrumentalists, there is a higher likelihood of injury following the elements of practice and performance that place increased demand on the neuromusculoskeletal system. More commonly, these could give rise to pathophysiological changes and, particularly, playing-related musculoskeletal disorders (PRMD’s).

The exact prevalence of PRMD’s in pianists is unclear; the majority of studies suggesting 40–65%, with an overall range of 26–93% (Bragge et al., 2006). This variability in range however, should be interpreted with caution. The definition of PRMD relating to pianists (and indeed any other instrumentalists) is not clear or consistent in the literature. Added to this, the varying methodological quality of the studies, it is not possible to clearly identify the prevalence of PRMD’s in pianists.

However, some considerable effort has been made to synthesize conflicting results in the literature. Bragge et al. (2006) performed a systematic review of prevalence and risk factors associated with PRMD’s in pianists. From a clinical perspective, Bragge, et al. highlighted that from the 12 studies that met their inclusion criteria, only four provided any operational definition of “injury” to identify cases of PRMD. These ranged from “Hand pain solely from playing-related overuse,” through to “Any problems caused by playing the piano which prevented piano playing for a period of 48 h or longer.” These descriptors serve to maximize the inclusion criteria of their respective studies, but do not provide consistent information to the prevalence of PRMD between each other. It is worth noting that the latter definition of 48 h or longer, provided in a study by Shields and Dockrell (2000), reported 25.8% suffered with symptoms of PRMD during the playing lifetime, or career, of the pianist. In a more recent study, Allsop and Ackland (2010) surveyed 505 pianists in Australia; 42.4% reported PRMD’s at the time when participating in the study (including the duration and location of symptoms), which aligned with the majority of studies reported by Bragge et al. (2006).

The highest ranked paper in the review by Bragge et al. (2006) was a survey and video study of 33 pianists (Yee et al., 2002). While their results indicate that 91% of participants had “a history of musculoskeletal symptoms,” all participants were measured with the clinical outcome SF-36 and all respondents were within normal ranges for that measure. Therefore any influence of PRMD at the time of measurement was retrospective. This study also highlighted the limited applicability of using broad inclusion criteria and the effect this has on skewing results. Thus, the prevalence of PRMD must be interpreted as “within the career” of a professional pianist, rather than at any one time.

The most prevalent pathologies that contribute to PRMD’s have been shown to be (Sakai, 2002):

• De Quervain’s tenosynovitis: affects the first extensor compartment that holds two tendons of the thumb; involved in extending and abducting the thumb. Symptoms include pain at the wrist and the base of the thumb, which are exacerbated during activity (Tubiana et al., 1996);
• Lateral epicondylitis (tennis elbow): affects the origin of the tendon for extensor carpi radialis brevis and also in 30% of cases, the anterior origin of extensor digitorum communis, both at the lateral epicondyle. This affects the movement of the forearm, particularly during pronation and supination, wrist movement and finger extension. Symptoms include pain at the elbow and are associated with strenuous overuse (Budoff, 2006);
• Focal dystonia: a neurological disorder affecting specific regions of the body. In musicians, it often manifests in the hand. Symptoms include involuntary movements, curling of the fingers, muscle weakness and tremor, and are associated with repetitive hand movements during playing (Butler, 2010).

These pathologies are commonly thought to relate generally to risk factors including excessive overuse, misuse, repetition of movements, and playing conditions (Lippman, 1991; Winspur, 2003; Furuya et al., 2006), which are often indicative of the high levels of practice and performance required of expert musicians.

Sakai (2002) investigated 200 consecutive overuse injuries related to piano performance and found pathologies divided into six main areas: tenosynovitis or tendinitis (56 cases), enthesopathy (49 cases), muscle pain (38 cases), neurological disturbance (28 cases), joint pain (24 cases), and neck or scapular pain (5 cases). Kjelland (2000) suggested that wrist movements during performance can fatigue forearm muscles, and therefore professional musicians can suffer from wrist injury. In the study by Sakai (2002), 35% reported the onset of symptoms, whilst playing an octave or chord, related to positions involving hyper abduction of the thumb and little finger in order to span the keys. However, a pianists’ hand span has not been shown to be a significant factor in symptoms of PRMD (Furuya et al., 2006).

The instrument played may also have an impact on the symptoms and locality of the PRMD. For example, pianists may exhibit symptoms relating almost exclusively to their wrists and hands (focal hand dystonia), whereas, due to required posture, other instrumentalists, such as flautists, are susceptible to neural or vascular impingement between the neck and the axilla (Sheibani-Rad et al., 2013). Violin and viola players may also experience nerve impingement in the neck and shoulder girdle (Berque and Gray, 2002). Level of expertise may also be an important factor in acquiring injury, for example expert pianists utilizing proximal joints to create passive forces at the wrist (Furuya and Kinoshita, 2008a), therefore minimizing the effort required at the distal finger joints.

In summary, common factors associated with impacts on health in performance science are:

• High levels of practice and repetition;
• Repetition with higher forces passing through individual joints, thus putting excessive pressure on surrounding tendons;
• Physical demands of musical pieces requiring joint movement at maximal limits of range, often coupled with higher forces.

## CONCLUSION

The benefits of using biomechanical methods for measuring musical performance have been discussed and the key findings described, highlighting and synthesizing contributions from the literature. Focusing on a bottom-up approach can further our understanding of motor control by utilizing biomechanical methods and principles. Applying biomechanical techniques to musical performance science is still in its infancy, and a fully integrated, comprehensive analysis has not yet been undertaken successfully. By combining biomechanical and neurophysiological techniques, and other methods such as auditory analysis, future studies will advance our understanding of the cognitive and neurophysiological mechanisms underpinning virtuosity during musical performance. The knowledge gained will highlight potential areas of future study, throughout the spectrum of functional ability from impairment, through to novice and expert, and on to virtuoso competencies in complex hand dexterity.

Many tools are available for use and, in principle, are appropriate for measuring complex hand dexterity. However, when applying biomechanical techniques, or indeed any measurement technique, the respective validity and reliability must first be defined. In addition to the confidence imparted through the use of valid and reliable measures, the measurement technique must not interfere with what is being measured. In abiding by these principles, biomechanical measurement can be used as a robust, accurate and reliable tool, as proponed by Bernstein, for furthering our understanding of the neurophysiological mechanisms underpinning learning, development of skill and complex hand dexterity.

## AUTHOR CONTRIBUTIONS

Cheryl D. Metcalf took overall responsibility for the production of the manuscript. Thomas A. Irvine and David O. Norris contributed to the manuscript specifically from the viewpoint of musical performance. Jennifer L. Sims contributed to the section on health considerations. Yu L. Wang and Alvin W. Y. Su contributed to the sections on EMG, EEG and imaging techniques. All authors were responsible for proof reading and contributing to the final draft of the manuscript.

## Conflict of Interest Statement

The authors declare that the research was conducted in the absence of any commercial or financial relationships that could be construed as a potential conflict of interest.
